# A Method for Real-Time Vessel Speed Measurement Based on M-YOLOv11 and Visual Tracking

**DOI:** 10.3390/s25133884

**Published:** 2025-06-22

**Authors:** Zhe Ma, Qinyou Hu, Yuezhao Wu, Wei Wang

**Affiliations:** 1 College of Merchant Marine, Shanghai Maritime University, Shanghai 201306, China; 202330110032@stu.shmtu.edu.cn (Z.M.); 202330110060@stu.shmtu.edu.cn (Y.W.); 2 Jiujiang Maritime Bureau, Jiujiang 332001, China; 15623990669@163.com

**Keywords:** vessel speed, target detection, visual tracking, machine visualization

## Abstract

In the context of vessel monitoring, the accuracy of vessel speed measurements is contingent on the availability of AIS data. However, the absence, failure, or signal congestion of AIS devices may lead to delays and inaccuracies in the speed information. To address this challenge, this paper proposes a vessel speed detection method based on target detection and tracking to acquire vessel speed in real time. The proposed methodology involves the establishment of a mapping relationship between image coordinates and four real-world coordinates, ensuring precise conversion from pixel velocity to physical velocity. Subsequently, a frame difference method combined with a multi-frame averaging strategy calculates the vessel speed. Furthermore, an advanced M-YOLOv11 detection model is introduced to enhance the detection performance in different vessel shapes and complex environments, thus ensuring the accuracy of speed information is further improved. The experimental results demonstrate that M-YOLOv11 exhibits a significant performance enhancement, with a 13.95% improvement in the average precision metric over the baseline model. Over 60% of the measured vessel speed measurement errors are less than 0.5 knots, with an overall average error below 0.45 knots. These findings substantiate the efficacy and superiority of the proposed method in practical applications.

## 1. Introduction

Shipping constitutes a pivotal component within the overarching framework of the global cargo transportation system [1,2]. In comparison with both land and air transport, shipping plays a role that is impossible to replace in the context of promoting regional economic growth and the rational allocation of mineral and other resources [3,4]. Its cost is low, and it can transport large volumes of goods. However, the effective monitoring and management of vessel speed is imperative to ensure the safety of waterways, maintain orderly shipping, and safeguard the ecological environment of the waters. Nevertheless, vessels frequently circumvent the maritime department’s regulations, particularly the speed limit guidelines, in the pursuit of enhanced transport efficiency, posing significant risks to vessel safety, the smooth operation of maritime channels, and the integrity of critical maritime infrastructure. This phenomenon not only substantially heightens the risk of vessel collisions but also has the potential to induce water disturbance, endangering the stability and safety of coastal infrastructure [5]. Currently, maritime authorities primarily rely on the speed information provided by vessels’ automatic identification systems (AIS) for regulatory purposes [6]. Nevertheless, there is a recognized challenge with AIS data due to its delay, malfunction, missing information, or vandalized systems, which can pose a significant hurdle when accurate, real-time speed information is required [7,8]. As a result, regulatory authorities face substantial difficulties in acquiring reliable, up-to-date speed data on vessels.This has led to the urgent necessity for the development of a cost-effective, widely applicable, easily deployable, and intuitively visualized real-time vessel speed measurement system. Such a system would greatly enhance the efficiency of water traffic supervision and ensure the safety of waterway operations.

In addition to AIS, maritime departments also use radar speed measurement and laser speed measurement technology to measure vessel speed. Despite the capability of these methods to provide real-time speed data for both incoming and outgoing vessels, practical applications encounter numerous limitations, hindering the ability to adequately address the demands of a complex and ever-changing shipping environment. For instance, radar speed measurement has the capacity to measure the speed of vessels over long distances [9]; however, its equipment cost is high, and it is susceptible to interference from environmental factors (such as obstacles), which can compromise the stability and reliability of the measurement [10,11]. Likewise, while laser speed measurement boasts superior accuracy, the cost of its equipment is high, and it is vulnerable to moisture damage [12]. External factors, such as fog on the water’s surface, can also compromise the accuracy of laser speed measurement, hindering its applicability in large-scale scenarios [13]. These challenges impede the existing speed measurement technology from meeting the maritime sector’s stringent requirements for accuracy, cost-effectiveness, and broad applicability in vessel speed monitoring. A thorough review of the extant literature reveals a paucity of studies addressing vessel speed measurement through the use of optical images. Moreover, there is an absence of a speed detection method that can adequately balance regulatory requirements and wide applicability. For instance, Broggi et al. [14] employ a monocular camera to capture video streams and integrate background subtraction, target tracking, and motion vector analysis to estimate vessel speeds using video frame rate and target baseline. In contrast, Andrade et al. [15] utilize a boundary tracking algorithm, in conjunction with water detection and the principle of uni-responsive transformation, to facilitate the calculation of vessel speeds. Zhao et al. [16] used an object detection model to identify vessels and applied the DeepSORT algorithm for trajectory tracking, ultimately converting pixel displacement into real-world displacement through camera calibration, and calculated vessel speed. Yan et al. [17] proposed a trajectory-aware NeRF framework capable of reconstructing 3D scenes from video, offering valuable insights into motion estimation and spatial structure learning from limited viewpoints. While these studies offer preliminary implementation solutions for vessel speed measurement, the limitations of environmental adaptability, algorithmic complexity, and dependence on camera calibration parameters impede its operability by non-specialists and its feasibility for practical applications.

Drawing upon extant research in the field, this paper puts forth a method for detecting vessel speed that is predicated on monocular vision and the coordinate transformation principle. The method utilizes surveillance cameras along the waterway to collect images of vessels and combines target detection and vision tracking technology to enable the real-time measurement of vessel speed. To enhance the adaptability and stability of the detection, this paper constructs a high-quality visible vessel dataset for the challenge of a complex water environment. It proposes a new feature extraction module, MDSC. It introduces the IP-IoU loss function to effectively improve the problems of insufficient target detection accuracy and poor detection frame stability in the tracking process. The remainder of the paper is structured as follows: Section II details the core process of the proposed method, including the specific steps of speed measurement, the design of the detection model, and the implementation of the visual tracking algorithm. Section III revolves around the experiments, including the experimental environment, the equipment parameter settings, and the experimental results and their analyses. Finally, Section IV concludes the research work of this paper.

## 2. Methodology

**Figure 1:**
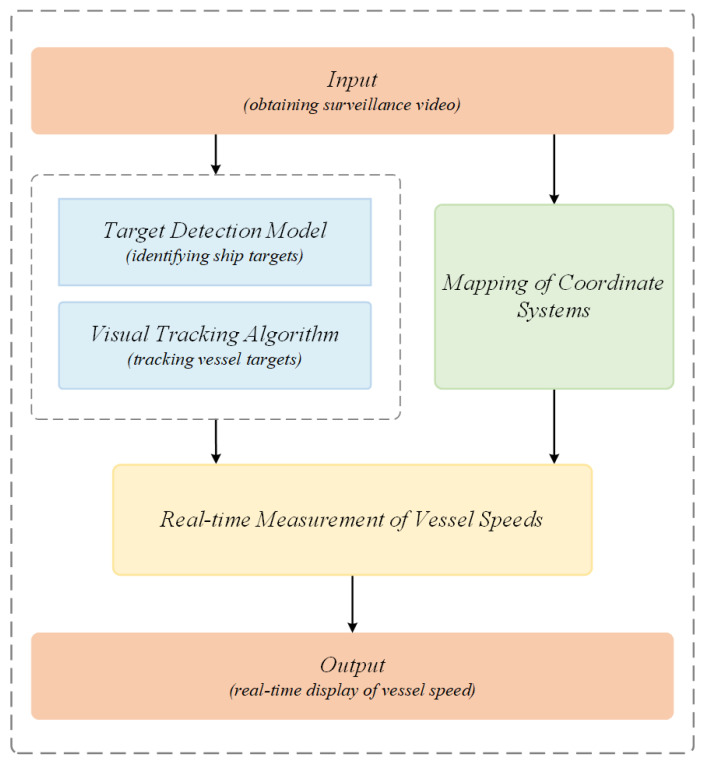
Overall workflow diagram of the system for vessel speed measurement. The video stream captured by the surveillance camera is processed in two ways, one is to detect and track the vessels in the frame, and the other is to establish a coordinate system projection and use it to obtain the necessary parameters. Finally, the speed of the vessel is measured according to the target detection frame information and parameters, and it is displayed on the monitor in real time.

The present section mainly focuses on the key technologies involved in vessel speed measurement, including the target detection model, vision tracking technology, coordinate system mapping, and camera calibration. Figure 1 presents the comprehensive workflow of the vessel speed measurement system. As illustrated, the system’s core components encompass the construction of a coordinate system and the calculation of speed. The accuracy and recognition rate of the target detection model directly impact the reliability of the speed measurement method. The stability of visual tracking technology is critical to ensure the accuracy of speed measurement for each vessel. Consequently, the judicious integration and optimization of these technologies is imperative to enhance the overall performance of the speed measurement system.

### 2.1. Targeted Detection

**Figure 2:**
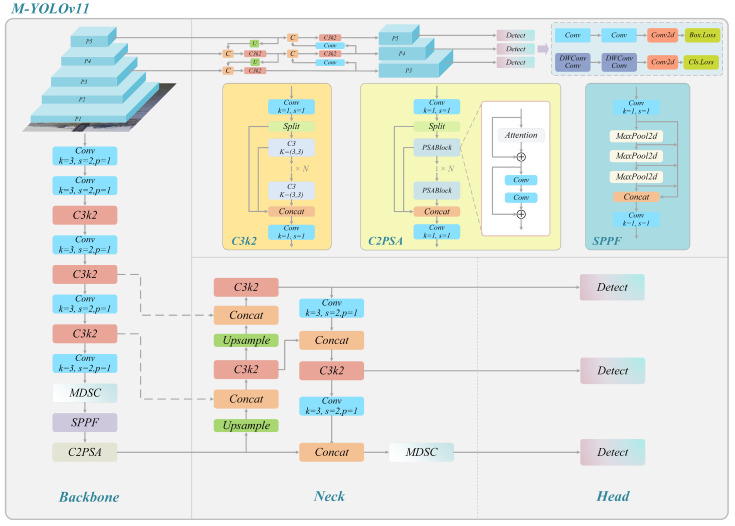
Overall structural framework of M-YOLOv11.

In the experimental process, this paper utilizes the surveillance camera provided by the maritime department for the target detection of vessels. The experimental findings demonstrate that the prevailing base model framework grapples with the challenges of suboptimal picture quality, target blurring due to fog interference, and missing feature information, thereby compromising the detection accuracy and robustness of the model. To address these challenges, this paper proposes an enhanced model based on YOLOv11 [18]. This model enhances the ability of the model to recognize vessel targets, reduces the leakage detection rate, and improves its adaptability in complex environments. Specifically, we propose the Multi-scale Depthwise Separable Convolution (MDSC) module to enhance the ability of the model to capture features of vessels of different scales and shapes, especially in the case of low picture quality, which can improve the accuracy of target detection. Furthermore, the IP-IoU (Interior Perception Intersection over Union) loss function is introduced to ensure that the model focuses on the consistency of the internal structure of the target with the bounding box, thereby enhancing the stability of the detection frame. This enhancement is paramount for the precise measurement of vessel speed, as the stability of the detection frame directly impacts the accuracy of the speed calculation. The integration of these two enhancements within the YOLOv11 framework results in the development of the M-YOLOv11 model, as depicted in Figure 2.

The M-YOLOv11 model comprises three distinct components as follows: Backbone, Neck, and Head [19]. The function of the Backbone component is principally to extract the fundamental feature information of the vessel from the image, thereby providing high-quality feature representations for the subsequent target detection [20,21]. The Neck component’s primary responsibility is to fuse the feature information extracted by the Backbone component, with the objective of enhancing the capacity of the model to detect targets at diverse scales [22]. This, in turn, leads to an augmentation of the model’s generalization performance in complex backgrounds. Finally, Head is responsible for the final classification and bounding box regression of the targets and outputs the detection results [23]. The following discussion will address both the MDSC module and the IP-IoU loss function.

#### 2.1.1. MDSC

**Figure 3:**
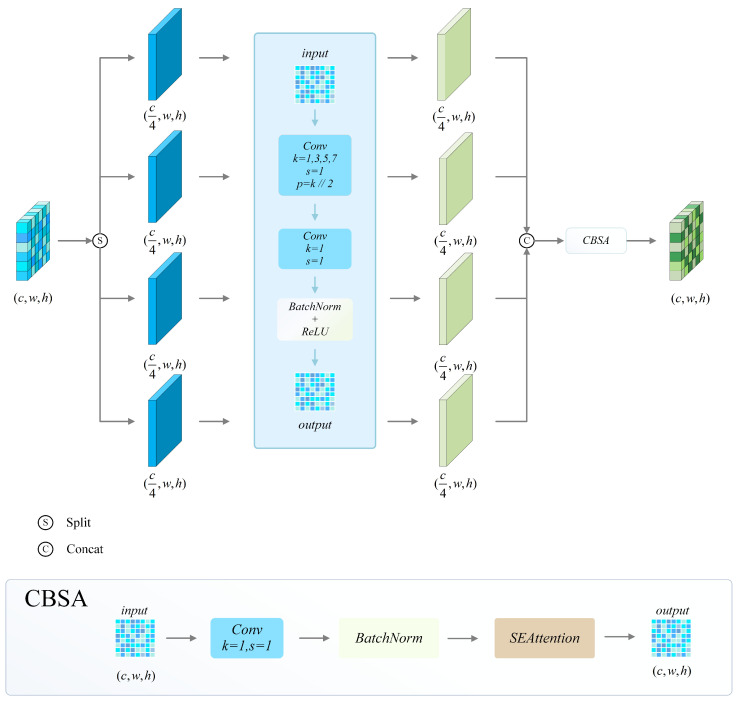
Structure and data flow of the MDSC module.

The MDSC module is composed of Multi-Scale Convolution, Depthwise Separable Convolution, and an SE (Squeeze-and-Excitation) attention mechanism. The module’s structure is shown in Figure 3. Multi-scale convolution extracts features at multiple scales by using convolution kernels with varying receptive fields. This enhances the ability of the model to adapt to different sizes of vessel targets and improves the generalization of detection [24,25]. The deep separable convolution enhances the model’s computational efficiency while reducing its computational complexity compared to standard convolution. This results in faster inference speeds while maintaining high detection accuracy [26]. The SE attention mechanism enhances the ability of the model to detect targets in complex environments by adaptively adjusting the weights of feature channels [27]. This highlights key features and suppresses redundant information, thereby improving the model’s performance.

In order to maximize the benefits of the MDSC module, this paper proposes a refined embedding method during experimentation. The experimental findings demonstrate that the extraction and integration of advanced semantic features can be efficiently enhanced by substituting the final C3k2 module in the Backbone and Neck structures with the MDSC module. This approach results in an enhanced model that significantly improves the capability to distinguish deep features and detect low-resolution vessel targets in challenging environments, such as water fog conditions. The optimization of information exchange [28] among multi-scale features establishes a balance between efficiency and performance, ensuring that the model remains both robust and suitable for deployment.

#### 2.1.2. IP-IoU

Due to the significant variations in vessel shape and size, as well as the complexity of inland waterway environments, traditional IoU can only reflect the overall overlap between the predicted and ground truth bounding boxes. However, it struggles to capture the fine-grained features and local structural information of vessel targets, which affects detection accuracy. To address this issue, this study introduces the Inner Powerful IoU (IP-IoU) loss function, enabling the predicted bounding box to not only outline the overall contour of the target but also assign higher importance to its internal key features. This enhancement effectively reduces localization errors caused by shape variations and environmental disturbances, thereby improving the model’s detection performance in complex scenarios. IP-IoU integrates and combines the strategies of Inner IoU [29] and Powerful IoU [30] to enhance the accuracy and robustness of object detection. Specifically, Inner IoU focuses on capturing fine-grained internal features of the target, ensuring that the detection model maintains high performance and stability even when the vessel undergoes shape distortion due to viewpoint variations or partial occlusion.

To effectively capture internal features, this study extracts the internal regions of the predicted bounding box $B_{p}$ and the ground truth bounding box $B_{g}$. Furthermore, the original bounding box is defined with its center point and dimensions as (x,y,w,h), where *x* and *y* represent the center coordinates, while *w* and *h* denote the width and height of the box, respectively. First, we introduce the scaling factor ratio, and we then calculate the boundary coordinates of the internal area as follows:

(1) $$\begin{array}{c} b_{x1}=x-\frac{w\times \mathrm{ratio}}{2},\;b_{x2}=x+\frac{w\times \mathrm{ratio}}{2}\\ b_{y1}=y-\frac{h\times \mathrm{ratio}}{2},\;b_{y2}=y+\frac{h\times \mathrm{ratio}}{2} \end{array}$$

At the same time, the IoU of the internal area is calculated, that is, the overlap between the predicted box and the internal area of the real box, as follows:

(2) $$\mathrm{inner}\_\mathrm{iou}=\frac{\mathrm{Area}(B_{p}^{inner}\cap B_{g}^{inner})}{\mathrm{Area}\left(B_{p}^{inner}\right)+\mathrm{Area}\left(B_{g}^{inner}\right)-\mathrm{Area}\left(B_{p}^{inner}\cap B_{g}^{inner}\right)+\varepsilon }$$

where $B_{p}^{inner}$ and $B_{g}^{inner}$ are the inner areas of the predicted box and the real box after scaling as above. ε is a very small constant.

The individual boundary coordinates of the predicted and real frames on the internal region are calculated according to Equation (1), respectively, as follows:

(a) $b_{1x1},b_{1x2},b_{1y1},b_{1y2}$: left, right, top, and bottom boundaries of the inner region of the prediction box.

(b) $b_{2x1},b_{2x2},b_{2y1},b_{2y2}$: left, right, top, and bottom boundaries of the inner region of the real box.

Subsequently, based on the calculated boundary coordinates, the deviations between the predicted box and the ground truth box in both the horizontal and vertical directions are computed to quantify their positional discrepancies as follows:

(3) $$\begin{array}{c} dw_{1}=\left|\min\left(b_{1x2},b_{1x1}\right)-\min\left(b_{2x2},b_{2x1}\right)\right|\\ dw_{2}=\left|\max\left(b_{1x2},b_{1x1}\right)-\max\left(b_{2x2},b_{2x1}\right)\right|\\ dh_{1}=\left|\min\left(b_{1y2},b_{1y1}\right)-\min\left(b_{2y2},b_{2y1}\right)\right|\\ dh_{2}=\left|\max\left(b_{1y2},b_{1y1}\right)-\max\left(b_{2y2},b_{2y1}\right)\right| \end{array}$$

The deviation is then normalized to obtain the position deviation value *P* as follows:

(4) $$P=\frac{1}{4}\left(\frac{dw_{1}+dw_{2}}{|w_{2}|}+\frac{dh_{1}+dh_{2}}{|h_{2}|}\right)$$

The deviation value P measures the distance between the predicted box and the true box in position. When the two are very close, the *P* value is small; otherwise, the *P* value increases. After obtaining *P*, the deviation of internal IoU and position can be calculated as follows:

(5) $$\mathrm{powerful}\_\mathrm{iou}=2-\mathrm{inner}\_\mathrm{iou}-\exp\left(-P^{2}\right)$$

At the same time, we define *q* and ensure that the smaller *P* is, the larger *q* is, and vice versa, as follows:

(6) q=exp(−P)

From Equation (6), we can further get the factor *x* that controls the gradient correction as follows:

(7) x=Λ·q

where Λ is used as an adjustment parameter to control the intensity of gradient correction.

In summary, combining the formulas, we can get the calculation method of IP-IoU as follows:

(8) $$IP\_IoU=1-3x\exp\left(-x^{2}\right)\cdot \mathrm{powerful}\_\mathrm{iou}$$

IP-IoU integrates internal feature matching and position correction strategies, enabling the predicted bounding box to more accurately align with the actual vessel target, thereby reducing prediction errors and improving detection accuracy. Moreover, it effectively handles vessel targets at varying distances and with diverse shapes, providing a solid foundation for accurate vessel speed detection in this study.

### 2.2. Visual Tracking

**Figure 4:**
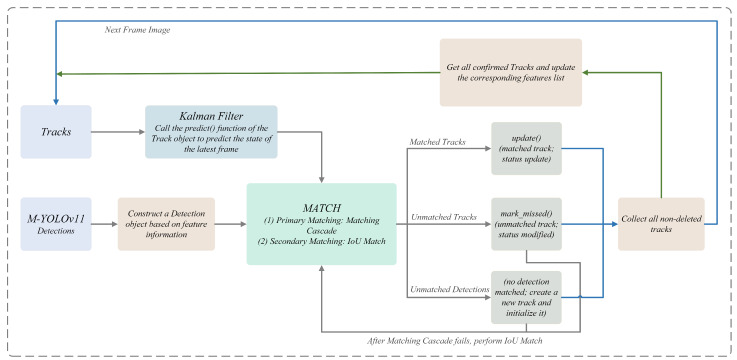
Example data stream for the DeepSORT algorithm.

The primary objective of visual tracking is to perpetually monitor and correlate the region of interest in the input video sequence to accurately ascertain its motion trajectory [31]. In this study, the DeepSORT algorithm is employed to track vessels, and its data flow is illustrated in Figure 4. DeepSORT integrates deep learning techniques with the original SORT algorithm, thereby enhancing the robustness and accuracy of tracking by extracting the target’s appearance features [32]. The core mechanisms of DeepSORT include a Kalman filter for target state prediction and a Hungarian algorithm for dynamic data association.

The Kalman filter is a state estimation algorithm that is optimal based on recursive estimation. It can predict the position of the target in the next frame [33]. Specifically, the Kalman filter employs the position, velocity, and other state variables of the vessel in the current frame to estimate the position of the target in the next frame through the state transfer equation [34]. It dynamically corrects the prediction results by combining them with observation data, thereby enhancing the continuity and stability of tracking. Furthermore, in order to address the issue of data correlation, DeepSORT employs the Hungarian algorithm to ensure optimal matching between the detected targets and the existing trajectories. The algorithm derives the similarity scores between disparate targets by constructing a cost matrix and makes the optimal allocation based on the principle of minimizing the global cost [34]. This process not only effectively reduces target confusion but also significantly reduces the ID Switch rate, thus improving the tracking accuracy and stability. The application of visual tracking technology establishes the foundation for subsequent vessel speed measurements. In the process of calculating speed by Frame Difference Method (FDM) [35], DeepSORT provides precise target trajectory information, thereby improving the reliability and accuracy of speed measurement results.

### 2.3. Speed Measurement

#### 2.3.1. Coordinate Transformation

**Figure 5:**
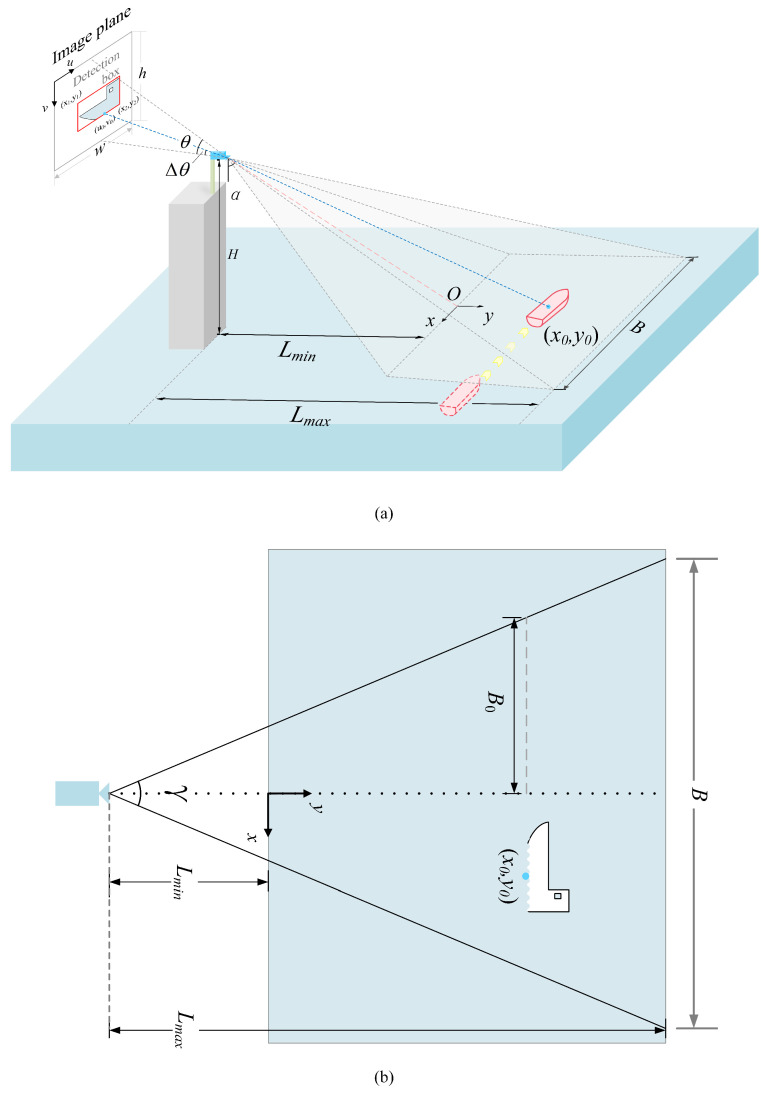
Schematic of the coordinate transformation plane. (**a**) Illustration of the camera projection model and geometric relationships between the image plane and the vessel’s position on the water surface. (**b**) Top-down schematic of the camera's field of view and the target vessel's position on the water surface.

To accurately calculate the vessel speed, it is essential to first determine the vessel’s actual position. Figure 5 illustrates the schematic diagram of the experimental environment under ideal conditions, with the relevant parameters for the coordinate transformation formula clearly labeled. Among these parameters, the camera height *H*, the actual width of the camera imaging top line *B*, the horizontal distance from the camera to the imaging bottom line $L_{\min}$, and the horizontal distance from the camera imaging top line $L_{\max}$ to the camera are fixed parameters that must be measured in the actual scene of the experiment in advance.

The pseudo-code presented in Table 1 offers a clear illustration of the fundamental process involved in coordinate transformation. Initially, rows 1, 2, and 3 in the table compute the three azimuths in the coordinate transformation system based on the tangent relationship of the trigonometric functions. Subsequently, it is hypothesized that the midpoint of the lower bottom edge of the detection box of the target ship detected by M-YOLOv11 $\left(u_{0},v_{0}\right)$ indicates the pixel position of the vessel. Point $\left(CEN\_X,y_{2}\right)$ represents the vessel’s pixel coordinates, calculated from the coordinates of the model’s output detection box. Here, $\left(x_{1},y_{1}\right)$ and $\left(x_{2},y_{2}\right)$ are the pixel coordinates of the upper-left and lower-right corners of the detection frame, respectively, as illustrated in the camera imaging plane shown in Figure 5a.

Afterward, the azimuth angle Δθ can be determined according to the proportionality relationship given by Equation (9) and the distance $y_{0}$ between the camera and the target vessel in the horizontal direction along the y-axis. The calculation process is detailed in Table 1, specifically, in rows 6 and 7.

(9) $$\frac{\Delta \theta }{\theta }=\frac{h-y_{2}}{h}$$

As illustrated in Figure 5b, the following relationship exists in the x-direction:

(10) $$\frac{\left|u_{0}-\frac{w}{2}\right|}{w}=\frac{\left|x_{0}\right|}{2\cdot B_{0}}$$

where $B_{0}$ denotes half of the pixel length of the vessel’s pixel coordinate dimension, which can be calculated using a trigonometric function, as detailed in row 7 of Table 1. Next, by utilizing the known parameters, we can derive the vessel’s coordinates in the x-axis direction, denoted as $x_{0}$. Based on this, the scale factor *e* can be further calculated, providing essential data for subsequent analysis and calculations. The calculation process is illustrated in rows 9 and 10 of Table 1.

#### 2.3.2. Vessel Speed Extraction

**Figure 6:**
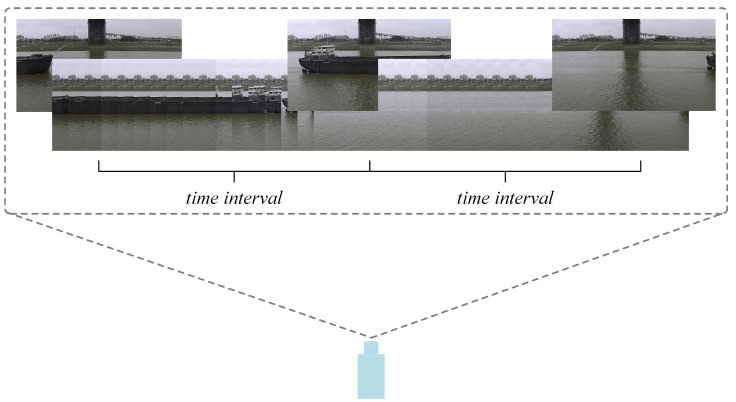
Method of framing differences.

Based on the coordinates of the vessel and the pixel scale factor *e* derived from the earlier coordinate transformation section, the actual speed of the vessel can be further calculated. We utilize the frame difference method to detect the speed of the vessel. As illustrated in Figure 6, the fundamental premise of this method is to use the time interval between two frames and the pixel displacement of the target to derive its actual speed. Specifically, the position of the vessel detection frame changes between two frames of images separated by a specific time interval. First, the vessel coordinates in both frames are recorded, and the pixel displacement is calculated. Subsequently, these pixel displacements are converted into real-world physical displacements using the scale factor *e*. Finally, by combining this information with the frame rate or inter-frame time interval, we can calculate the velocity of the vessel’s motion in physical space. The specific algorithmic flow for velocity calculation is presented in the pseudo-code in Table 2.

The pseudo-code delineates the fundamental process for calculating vessel speed. Initially, a nested loop traverses all detected vessel bounding boxes in the first and second frames to extract their coordinate values. These coordinates are then input into the algorithm described in the coordinate transformation section to obtain the actual coordinates ($x_{0}$, $y_{0}$), ($x_{0}^{\prime }$, $y_{0}^{\prime }$) of the target vessel in the two frames, respectively. The subsequent step involves calculating the actual displacement of the vessel using the Euclidean distance formula. This displacement is then multiplied by a scale conversion factor *e* and divided by the time interval *t* between the two frames to obtain the actual velocity in meters per second (m/s). Finally, the calculated velocity must be converted to the standard unit of measurement (knots) for practical applications, as demonstrated in rows 4–6 of Table 2. To enhance the precision of the calculations, multiple speed measurements of the same vessel are taken; outliers are identified and eliminated, and the remaining measurements are averaged to minimize errors.

### 2.4. Camera Calibration

**Figure 7:**
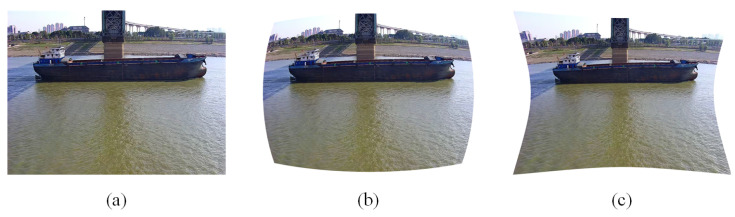
The following figures illustrate the image distortion and the image after correction. As illustrated in Figure (**a**), the image has been corrected, while Figure (**b**) depicts the barrel distortion image and Figure (**c**) shows the occipital distortion image.

In the domain of machine vision, camera image distortion has been identified as a primary factor that can compromise the precision of vision computation [36]. Distortions such as barrel distortion and pincushion distortion are frequently observed. Barrel distortion, a prevalent issue in wide-angle lenses, manifests as a relative enlargement of the image center area and an outward expansion of the edge area, resulting in the appearance of straight lines as convex arcs [37]. Pincushion distortion, another common type of distortion, is characterized by the inward contraction of the image edges, resulting in a distortion of the image resembling a pillow. Figure 7 provides a visual representation of the image that has undergone calibration and correction, as well as the image that has been distorted without correction.

The paper posits that accurate measurement of vessel speed is contingent on the precise mapping between the pixel coordinate system and the world 2D coordinate system. Camera distortion has been shown to compromise the correspondence between image pixels and the physical scale, thereby influencing the accuracy of speed calculations. To ensure the accuracy of vessel speed measurement, this paper adopts the Checkerboard Calibration Method (CCM) to calibrate the surveillance camera [38,39]. This method compensates and corrects the distortion problem, thereby improving the measurement accuracy and robustness of the system.

## 3. Experiments and Results

### 3.1. Experimental Environment

In this paper, the Jiujiang River Basin of the Yangtze River is selected as the research scenario. Speed measurement experiments of vessels passing through the channel are carried out by relying on the monitoring camera and the basic computing equipment set up at the Jiujiang Bridge of the Yangtze River. The specific technical specifications of the equipment utilized are enumerated in Table 3, thereby providing a reference framework for the experimental repeatability and the system deployment.

### 3.2. Vessel Target Detection

#### 3.2.1. Datasets and Experimental Setup

**Figure 8:**
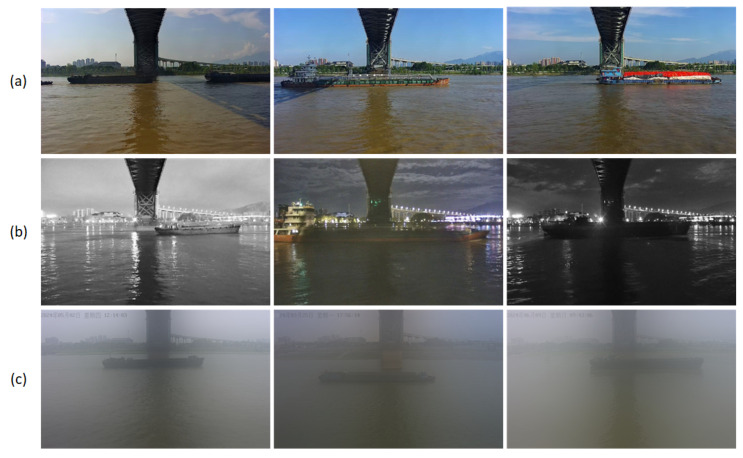
Vessel dataset presentation. Group (**a**) shows daytime images, group (**b**) shows nighttime images, and group (**c**) shows images in foggy scenarios.

In order to enhance the recognition accuracy and robustness of the detection model for vessel targets, and to address the actual needs of the maritime department in complex application scenarios, this paper proposes the construction of a high-quality visible vessel dataset. This dataset is based on surveillance cameras along the waterside, which collect images of vessels in different time periods and under different weather conditions, as illustrated in Figure 8. The dataset encompasses 3700 vessel images, encompassing typical scenes such as daytime, nighttime, and dense fog, thereby enhancing the model’s adaptability in complex environments. During the model training process, the dataset is divided into a training set, a validation set, and a test set according to a ratio of 7:2:1. This ensures the adequacy of the model training and the objectivity and reliability of the performance evaluation.

In this paper, the same parameter settings are adopted in the ablation and comparison experiments of the improved M-YOLOv11 to ensure the comparability of the experimental results so as to accurately assess the effectiveness of the improved module and the performance difference between different models. A comprehensive list of the key experimental parameters is provided in Table 4.

#### 3.2.2. Model Evaluation Indicators

In order to evaluate the detection performance of M-YOLOv11 on vessel targets in various environments, this paper utilizes the same evaluation metrics in the ablation and comparison experiments, which include recall, average precision (AP), parameters, and floating point operations (FLOPs).

Recall is defined as the ratio of correctly detected positive samples to the total number of actual positive samples, given as follows:

(11) $$\mathrm{Recall}=\frac{TP}{TP+FN}\;$$

where TP represents the number of correctly detected positive samples, and FN denotes the number of positive samples that were missed.

AP is used to evaluate the target detection accuracy of the model at different confidence thresholds, which is actually expressed as the area under the precision–recall curve as follows:

(12) $$\mathrm{AP}=\int_{0}^{1}P\left(R\right)dR\;$$

where P(R) represents the precision–recall curve, and dR denotes the recall increment.

Params refers to the total count of trainable parameters in the model, and it is often used to measure model complexity as follows:

(13) $$\mathrm{Params}=\sum_{l=1}^{L}\left(C_{\mathrm{in}}\times C_{\mathrm{out}}\times K^{2}+C_{\mathrm{out}}\right)$$

where *L* is the number of layers, $C_{in}$ and $C_{out}$ denote the input and output channels per layer, and *K* is the kernel size.

FLOPs are used to measure the amount of computation required for model inference, which is specifically defined as follows:

(14) $$\mathrm{FLOPs}=\sum_{l=1}^{L}\left(2\times C_{\mathrm{in}}\times C_{\mathrm{out}}\times K^{2}\times H\times W\right)$$

where *H* and *W* are the height and width of the feature map.

#### 3.2.3. Ablation Experiments

**Figure 9:**
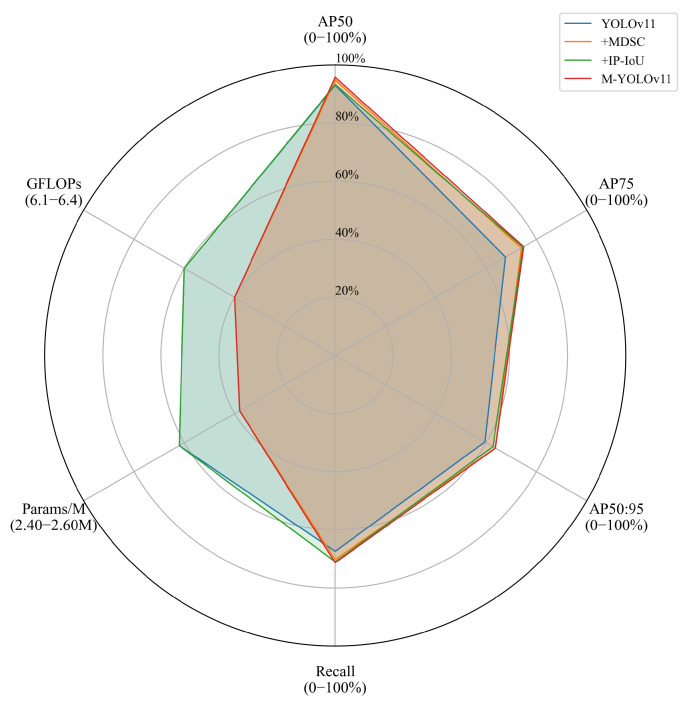
Radar diagram of the ablation experiment data.

In the context of this study, we employed YOLOv11 as the baseline model for our ablation experiments. We systematically introduced various enhancement modules to assess the impact of each module on the efficacy of vessel target detection. The experimental findings are presented in Table 5 and Figure 9, which illustrate the impact of various enhancement schemes on the detection accuracy and model performance.

Both from the tabular data and the radar diagrams shown in Figure 9, it can be observed that compared with YOLOv11, the improved M-YOLOv11 in this paper realizes a significant improvement in all performance indicators. Specifically, the AP _50_ score increased from 93.0% to 95.7%; the AP _75_ score increased from 67.7% to 74.9%, which represents an increase of over 7%; and the most stringent composite metric, ${\mathrm{AP}}_{50:95}$, increased from 59.5% to 63.7%. A detailed analysis reveals that MDSC enhances target detection capability across various scales through multi-scale feature fusion, contributing to a 6.7% increase in ${\mathrm{AP}}_{50:95}$. In contrast, IP-IoU optimizes target boundary regression, leading to a 6.9% rise in ${\mathrm{AP}}_{75}$. The radargram further validates the optimization of M-YOLOv11 in terms of computational complexity. A clear observation reveals that both the GFLOPs and Params metrics of the model are closer to the center of the circle. This indicates that while enhancing the detection performance, it does not escalate the computation cost, and rather, it curtails the parameter scale to a certain extent. This outcome substantiates the efficacy of the enhanced methodology outlined in this study, underscoring its substantial enhancement of the model’s detection precision while preserving its operational efficiency. This finding is of paramount significance, as it underscores the model’s potential for practical applications.

#### 3.2.4. Comparative Experiments

To validate the effectiveness of M-YOLOv11, we adopted state-of-the-art evaluation metrics and methods to comprehensively assess the detection accuracy and computational complexity of models such as the YOLO series, EfficientDet, and RetinaNet through a series of comparative experiments. The results of the comparative experiments for each model are shown in Table 6.

The experimental results demonstrate that M-YOLOv11 outperforms all other YOLO series models across various evaluation metrics, further validating the effectiveness of the proposed improvements. Specifically, M-YOLOv11 achieves an ${\mathrm{AP}}_{50}$ of 95.7%, representing an increase of 21.0%, 4.4%, and 1.5% compared to YOLOv3, YOLOv5, and YOLOv8, respectively. In terms of the ${\mathrm{AP}}_{75}$ metric, M-YOLOv11 attains 74.9%, surpassing YOLOv3, YOLOv5, and YOLOv8 by 31.6%, 11.8%, and 9.6%, respectively. Moreover, for the most stringent ${\mathrm{AP}}_{50:95}$ metric, M-YOLOv11 achieves 63.7%, which is 22.4% higher than YOLOv3 and also outperforms YOLOv5 and YOLOv8 by 5.9% and 4.0%, respectively. In terms of recall, M-YOLOv11 reaches 71.3%, surpassing YOLOv3, YOLOv5, and YOLOv8 by 24.8%, 4.8%, and 2.4%, respectively.

Additionally, we compared it with models outside the YOLO series. The results showed that although EfficientDet has advantages in terms of computational complexity and parameter count, its ${\mathrm{AP}}_{50}$, ${\mathrm{AP}}_{75}$, and ${\mathrm{AP}}_{50:95}$ values are significantly lower than those of the YOLO series, and its recall rate is only 49.1%, making it difficult to meet the requirements for high-precision detection. The RetinaNet model performs reasonably well in terms of detection accuracy; however, its high computational overhead of 122.9 GFLOPs and parameter count of 19.78 million result in prohibitively high deployment costs in practical applications. Notably, despite these significant performance improvements, M-YOLOv11 maintains a smaller parameter count (2.46 M params) and computational cost (6.2 GFLOPs) among all compared models, further demonstrating that the proposed method enhances detection accuracy and robustness while ensuring computational efficiency.

**Figure 10:**
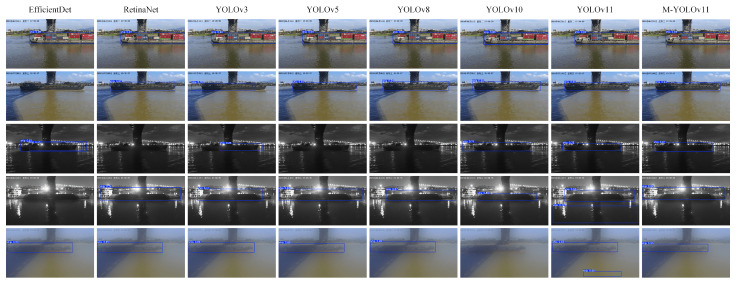
Performance of different models for the detection of vessels.

As illustrated in Figure 10, the quantitative detection results of each model on the test set are presented. It is evident from the figure that, in daylight scenes with adequate lighting, all models can attain more optimal detection outcomes due to the distinct texture of the vessel. Conversely, in scenarios characterized by low illumination or poor visibility, such as nocturnal conditions or foggy days, the enhanced M-YOLOv11 demonstrably surpasses its comparative counterparts. This superiority is evident in metrics including target recognition accuracy, the confidence level of the detection frame, and the alignment of the bounding box with the target. In nighttime scenes, the other models frequently failed to detect the target or exhibited incomplete coverage of the detection frame. In contrast, M-YOLOv11 demonstrated enhanced robustness and environmental adaptability.

### 3.3. Experiments on Speed Measurements

This section delineates the outcomes and shortcomings of the vessel speed measurement experiments. These outcomes are subsequently analyzed.

#### 3.3.1. Measurement Results

**Figure 11:**
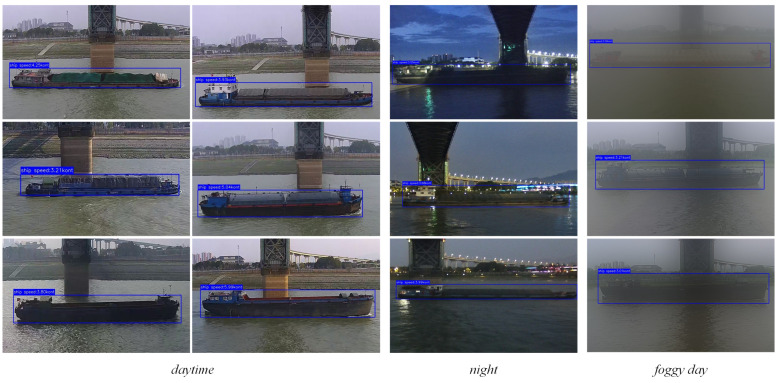
Real-time measurement of vessel speed at different times of the day and in different weather conditions.

In order to verify the validity and accuracy of the vessel speed measurement method proposed in this paper, the surveillance cameras in the Jiujiang section of the Yangtze River Basin were utilized to randomly select 100 vessels in the channel for speed measurement and record their measurement results. The experiments encompass a range of vessel types, including bulk carriers and liquid cargo vessels. Samples from different time periods and weather conditions are also selected to ensure that the method is applicable to a wide range of actual shipping environments, so as to comprehensively evaluate its robustness and applicability. As illustrated in Figure 11, the monitoring screen displays the real-time vessel speed measurement, presenting the detection outcomes and the associated speed data of the vessel.

**Figure 12:**
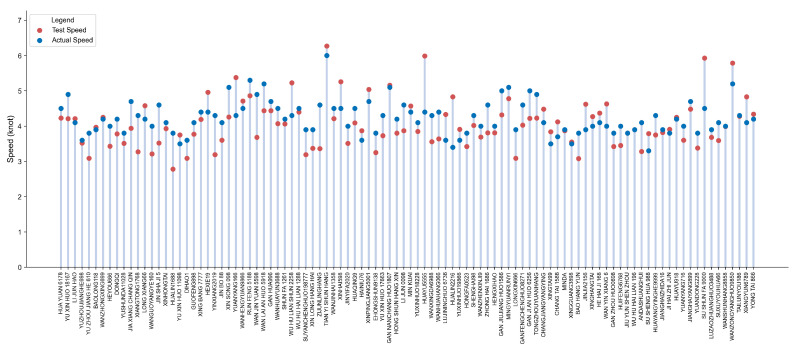
Lollipop chart comparing experimental data of vessel speed detection. Each vessel in the chart corresponds to a lollipop, and the red and blue balls on each lollipop represent the test speed and actual speed of the corresponding vessel, respectively.

The AIS receiver is employed to obtain the AIS data of each vessel, and the true speed of the vessel is parsed out from it. Subsequently, the speeds obtained based on the visual measurement method were compared and analyzed with the real speeds recorded by AIS, and a lollipop plot of the speed comparison was drawn (as shown in Figure 12). As depicted in Figure 12, the measured speeds of the 100 vessels tested exhibited a strong correlation with the actual speeds recorded by AIS, with only a few vessels demonstrating significant deviations.

The speed distribution of the 100 vessels is summarized in Table 7. The results indicate that the maximum speed of the tested vessels did not exceed 7 knots, with more than half of the vessels’ speeds falling within the range of 3–4 knots. This outcome indicates that the vessels in this particular waterway predominantly adhere to the established speed regulations set forth by the local maritime authorities. This outcome serves to substantiate the precision and dependability of the vision-based vessel speed measurement technique outlined in this study, underscoring its significant practical value in the domain of shipping regulation.

#### 3.3.2. Inaccuracy Analysis

We take the absolute value of the difference between the test speed and the actual speed of 100 vessels to calculate the measurement error of vessel speed, perform interval statistics on the error value, and finally obtain the relationship between the error interval and the number of vessels, as shown in Table 8. The results indicate that, among the 100 vessels examined, over 60% exhibited speed measurement errors less than 0.5 knots, approximately 25% demonstrated errors ranging from 0.5 to 1.0 knots, and only a limited number of vessels exhibited errors exceeding 1.0 knot. It is noteworthy that in this experiment, the speed measurement error for two vessels was recorded as 0.

**Figure 13:**
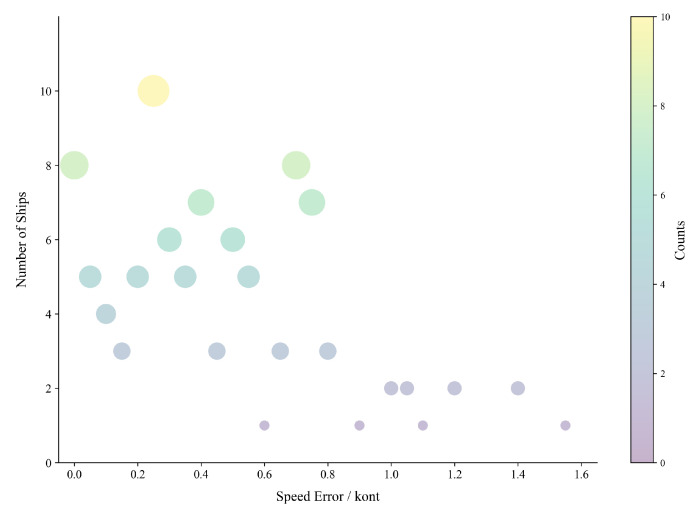
Distribution of speed error and number of vessels.

In order to present the error distribution in a more intuitive manner, a bubble diagram was constructed to illustrate the speed measurement errors of vessels (see Figure 13). The figure reveals that the majority of the bubbles, corresponding to the number of vessels, are concentrated on the left side of the figure. This observation indicates that the speed measurement errors of the majority of vessels are minimal, consistent with the error statistics enumerated in Table 8.

In practical applications, reduced visibility caused by water mist may lead to errors in vessel speed measurements. Based on the 100-speed measurement data in Section 3.3.1 above, and combined with the visibility information provided by the meteorological station in the Jiujiang section of the Yangtze River basin, we analyzed the measurement errors of the method under different visibility conditions, as shown in Table 9. The results show that when visibility is greater than 200 m, the measurement impact caused by visibility is negligible, and the speed error of most vessels is controlled within ±1 knot. When visibility ranges from 50 m to 200 m, we benefit from the improved detection model’s robustness to foggy images, and speed errors remain at a low level. However, under the meteorological bureau’s defined "extremely dense fog" conditions (visibility below 50 m), due to significant declines in camera imaging quality and detection accuracy, speed measurement errors increase markedly.

## 4. Conclusions

In this paper, a real-time vessel speed measurement method based on machine vision is proposed for the needs of vessel speed measurement and supervision in the key areas of waterways by the maritime department. The proposed method integrates target detection and vision tracking techniques, leveraging the mapping relationship between pixel coordinates and world coordinates to achieve the precise measurement of vessel speed. To enhance the target recognition accuracy of this method in complex environments, this paper proposes the MDSC module and introduces the IP-IoU loss function. This function is integrated into the existing model and optimized as M-YOLOv11, thereby outperforming the comparison model in all performance indexes. The experimental results demonstrate that among the 100 vessels selected at random for testing, over 60% exhibited a speed measurement error of less than 0.5 knots, more than 90% demonstrated an error of less than 1 knot, and the overall average error did not exceed 0.45 knots. These findings substantiate the efficacy and reliability of the proposed method in measuring vessel speed and underscore its significant practical value.

## Figures and Tables

**Table 1:** Pseudo-code for the coordinate transformation and calculation of scale factor e.

Pseudo-Code	Line
FUNCTION CT():	
SET α = arctan($L_{\min}$ / *H*)	1
SET θ = arctan($L_{\max}$ / *H*)-α	2
SET γ/2 = arctan(B/2 / $L_{\max}$)	3
FOR EACH (x1, y1, x2, y2) IN DETECTION_BOX DO:	
SET CEN_X = (x1 + x2) / 2	4
SET CENTER_DISTANCE = | CEN_X-(*w* / 2) |	5
SET Δθ = ((*h* - y2) * θ) / *h*	6
SET $y_{0}$ = *H* * tan(α + Δθ) - $L_{\min}$	7
SET $B_{0}$ = ($y_{0}$ + $L_{\min}$) * tan γ/2	8
SET $x_{0}$ = (2 * $B_{0}$ * | CEN_X - (*w* / 2) |) / *w*	9
SET e = CENTER_DISTANCE / $x_{0}$	10
ENDFOR	
END FUNCTION	

**Table 2:** Pseudo-code for the vessel speed calculation process.

Pseudo-Code	Line
FUNCTION SPEED():	
FOR EACH ($x_{0}$, $y_{0}$) IN VESSEL1 DO:	1
FOR EACH ($x_{0}^{\prime }$, $y_{0}^{\prime }$) IN VESSEL1* DO:	2
// Calculate vessel speed using scaling factor ‘*e*’	3
SET VESSEL_SPEED = *e* * $\sqrt{{\left(x_{0}-x_{0}^{\prime }\right)}^{2}+{\left(y_{0}-y_{0}^{\prime }\right)}^{2}}$ / *t*	4
// Convert speed from m/s to knot	5
SET VESSEL_SPEED = VESSEL_SPEED * 1.94384	6
ENDFOR	7
ENDFOR	
END FUNCTION	

**Table 3:** Parameters of the experimental equipment.

Scope	Numeric
OS	Ubuntu 22.04
CPU	Intel Xeon E5 2680v4
GPU	Tesla-P40
Memory	6 GB
Graphics Memory	24 GB
Programming Language	Python 3.10.15
Deep Learning Frameworks	Pytorch 2.3.0
CUDA	12.1
Monitor	Hikvision Spherical Camera

**Table 4:** Prescribed parameters for model training.

Scope	Numeric
Optimizer	Adam
lr0	0.0001
lrf	0.01
Image Size	640 × 640
Epoch	100
Batch Size	16
Worker	6
Momentum	0.9

**Table 5:** Results of the ablation experiments.

Model	AP_50_/%	AP_75_/%	AP_50:95_/%	Recall/%	Params/M	GFLOPs
YOLOv11	93.0	67.7	59.5	67.4	2.58	6.3
+ MDSC	94.8	74.0	63.7	70.2	2.46	6.2
+ IP-IoU	93.2	74.6	62.7	71.0	2.58	6.3
M-YOLOv11	95.7	74.9	63.7	71.3	2.46	6.2

**Table 6:** Performance parameters of the model in ablation experiments.

Model	AP_50_/%	AP_75_/%	AP_50:95_/%	Recall/%	Params/M	GFLOPs
EfficientDet	64.3	43.1	41.0	49.1	3.83	4.7
RetinaNet	89.7	55.4	46.5	58.7	19.78	122.9
YOLOv3	74.7	43.3	41.3	46.5	12.13	18.9
YOLOv5	91.3	63.1	57.8	66.5	2.50	7.1
YOLOv8	94.2	65.3	59.7	68.9	3.01	8.1
YOLOv10	67.5	43.4	40.5	46.8	2.69	8.2
YOLOv11	93.0	67.7	59.5	67.4	2.58	6.3
M-YOLOv11	95.7	74.9	63.7	71.3	2.46	6.2

**Table 7:** Vessel test speed intervals and number of vessels.

Speed Range (Knots)	Number of Vessels
speed < 1.00	0
1.00 ≤ speed < 2.00	0
2.00 ≤ speed < 3.00	1
3.00 ≤ speed < 4.00	53
4.00 ≤ speed < 5.00	36
5.00 ≤ speed < 6.00	9
6.00 ≤ speed < 7.00	1
speed ≥ 7.00	0

**Table 8:** Quantitative statistics under specified error intervals.

Error Range (Knots)	Number of Vessels
speed = 0.0	2
0.0 < speed ≤ 0.1	2
0.1 < speed ≤ 0.3	31
0.3 < speed ≤ 0.4	25
0.5 < speed ≤ 0.7	18
0.7 < speed ≤ 0.9	12
0.9 < speed ≤ 1.1	4
1.1 < speed ≤ 1.3	3
1.3 < speed ≤ 1.5	2
1.5 < speed ≤ 1.7	1

**Table 9:** Errors under different visibility conditions.

Error Range (Knots)	Visibility Level
0.0 ≤ error < 0.4	≥200 m
0.4 ≤ error < 1.0	100–200 m
1.0 ≤ error < 1.6	50–100 m
error ≥ 1.6	<50 m

## Data Availability

The datasets analyzed or generated in this study are available from the authors upon reasonable request.
